# Solid Pseudopapillary Tumor of the Pancreas in a 50-Year-Old Man: A Case Report and Review of the Literature

**DOI:** 10.1089/crpc.2016.0002

**Published:** 2016-04-01

**Authors:** Xie Hongjian, Li Dujuan, Xue Shuang, Zhao Yuewu, Kong Lingfei

**Affiliations:** Department of Pathology, Henan Provincial People's Hospital, People's Hospital of Zhengzhou University, Zhengzhou, People's Republic of China.

**Keywords:** solid pseudopapillary tumor, pancreatic tumor, pancreatic neoplasm

## Abstract

***Background:*** A solid pseudopapillary tumor (SPT) of the pancreas is a rare neoplasm with low malignancy, constituting about 2% of pancreatic tumors, which mainly occurs in young women.

***Case Presentation:*** We herein report a case of a small SPT arising from the head of the pancreas in an asymptomatic 50-year-old man. This patient was admitted to our department at Henan Provincial People's Hospital for the evaluation of a pancreatic mass and a pancreatic resection was performed. Histology revealed the lesion to be an SPT of the pancreas, with the characteristic pseudopapilla formation, central degeneration, and capsule formation. The tumor was positive for vimentin, CD10, α1-antichymotrypsin, α1-antitrypsin, β-catenin, neuron-specific enolase, synaptophysin, and progesterone receptor.

***Conclusion:*** We diagnosed an SPT in the patient based on these histological findings and immunophenotype.

## Introduction

Solid pseudopapillary tumors (SPTs) of the pancreas, first described by Virginia Frantz in 1959,^1^ are an uncommon but distinct pancreatic neoplasm with low-grade malignant potential, accounting for 0.2–2.7% of all pancreatic tumors.^2^ They typically occur in the body or tail of the pancreas and predominantly affect young females.^3^ In 1996, the World Health Organization renamed it as SPT, which has a variety of names including solid and cystic tumor, Frantz tumor, papillary epithelial neoplasm, solid-cystic papillary epithelial tumor, and papillary cystic tumor. Pathological examination reveals that SPTs are usually a large, encapsulated mass composed of a mixture of cystic, solid, and hemorrhagic components. Both a capsule and intratumoral hemorrhage are important clues to the diagnosis because these features are rarely found in other pancreatic neoplasms. SPTs generally occur in young women and are rarely reported in middle-aged or elder males. In this article, we report a case of a 50-year-old man who presented with a small SPT in the area of the pancreatic head with clinical information, computed tomography (CT) findings, pathological features, and a literature review.

## Case Report

**FIG. 1.**
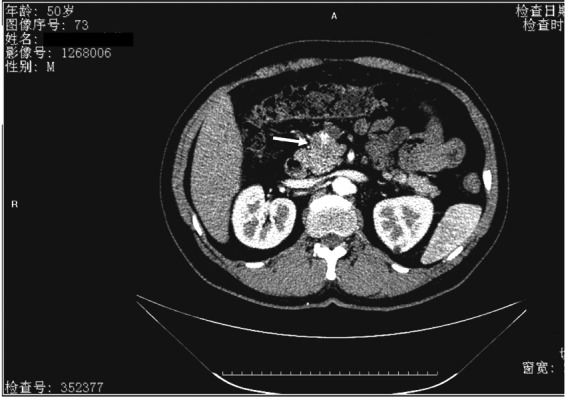
CT scan of a patient with an SPT. Abdominal contrast-enhanced CT scan shows an enlarged pancreatic head containing a well-defined, encapsulated solid cystic mass about 4 cm in diameter (arrow). CT, computed tomography; SPT, solid pseudopapillary tumor.

A previously healthy 50-year-old man was admitted to Henan Provincial People's Hospital with a month's history of unspecific epigastric abdominal discomfort. This patient had no significant history of medical or alcohol abuse and hospitalization. Physical examination and laboratory tests were unremarkable. Abdominal ultrasonography (USG) and CT scan showed an enlarged pancreatic head containing an uneven mixed solid and cystic composition about 3.7 × 4.0 cm (Fig. 1). No surrounding lymphadenopathy was noted. An underlying neoplasm was suspected and these findings suggested a solid pancreatic head neoplasm.

**FIG. 2.**
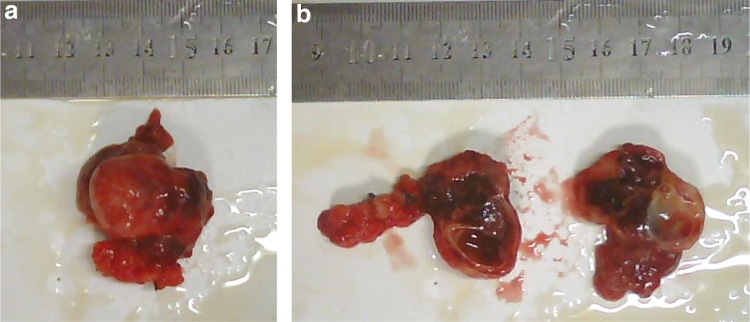
Gross examination of an SPT. Photograph of the **(a)** gross specimen shows the **(b)** smoothly encapsulated tumor with areas of necrosis and hemorrhage. The ruler shows distance in centimeters.

**FIG. 3.**
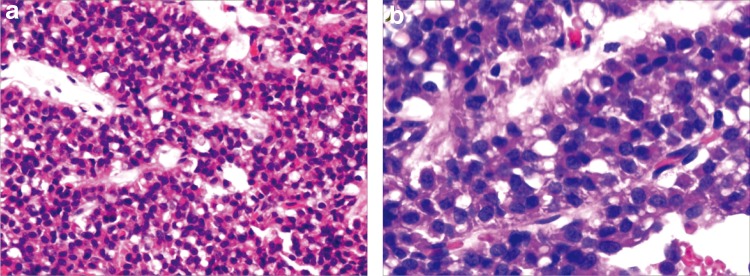
Staining with hematoxylin and eosin shows histopathological appearance of an SPT. The tumor was histologically composed of uniform polygonal cells arranged in the form of solid sheets, microcysts, and pseudopapillary areas filled with hemorrhage and necrotic debris (hematoxylin and eosin stain, magnification in **a**: ×20; magnification in **b**: ×40).

**FIG. 4.**
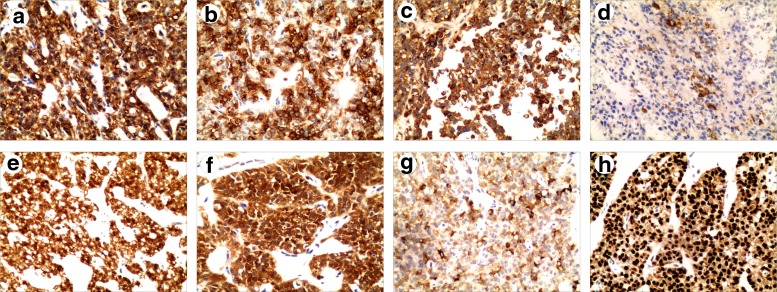
The immunohistochemical features of an SPT. The tumor cells showed positive for **(a)** vimentin, **(b)** CD10, **(c)** α-antichymotrypsin, **(d)** α-antitrypsin, **(e)** β-catenin, **(f)** neuron-specific enolase, **(g)** synaptophysin, and **(h)** progesterone receptor.

The patient underwent enucleation of the neoplasm in the pancreatic head and was sent to the pathology department. On gross examination of the surgical specimen, a soft, round, well-circumscribed mass with pseudocapsule about 3.6 cm in diameter was identified (Fig. 2). It had pale brown or grayish white solid portion, papillary projections, and cystic portions resulted from hemorrhagic necrosis. On histological analysis, the tumor was composed of monotonous uniform polygonal cells with moderate to abundant amphophilic cytoplasm and arranged in solid nests with areas of cystic degeneration, characterized by separation of the cells into pseudopapillary aggregates with intervening accumulation of mucopolysaccharide-rich ground substance (Fig. 3). No vascular space or peripheral invasion was identified. The tumor was encapsulated and although the tumor–pancreatic parenchyma interface was irregular on histological analysis, the tumor did not invade the pancreas. The tumor cell nuclei were oval or coffee bean shaped (Fig. 3). No mitotic figures were identified in 20 HPF (4.75 mm^2^) from various areas of the tumor. On immunohistochemical analysis, the tumor cells were positive for vimentin, CD10, α-1-antichymotrypsin (AACT), α-1-antitrypsin (AAT), β-catenin, neuron-specific enolase (NSE), progesterone receptor (PR), and synaptophysin (Syn) in approximately 30% of the cells, whereas cells were positive for Ki-67 antigen (a proliferation marker) less than 1% of the neoplastic cells (Fig. 4). These findings helped to establish a diagnosis of SPT of the pancreas. Six months after surgery, a repeat CT scan of the pancreas revealed no evidence of the tumor. The patient is still alive at the time of publication.

## Discussion

SPTs of the pancreas are a relatively uncommon pancreatic neoplasm of uncertain origin. These tumors have a strong tendency to appear primarily in young women during the second to fourth decade of life, and they are histologically characterized by cystic areas and solid pseudopapillary arranged cells. Although SPTs may affect any portion of the pancreas, they are more common in the pancreatic body or tail.^4,5^ These neoplasms account for 5% of cystic pancreatic tumors and 1–2% of exocrine pancreatic neoplasms.^5^ SPTs have low-grade malignant potential with invasion or metastasis in 15%, involving vascular and nerve sheath invasion or lymph node and liver metastases.^6^ Local recurrences have rarely been reported. Overall, 5-year survival is as high as 97% in patients undergoing surgical resection, even in the presence of extension into adjacent organs or metastases.^7^ Previous studies identified that SPTs have more malignant potential when the lesion occurs in elderly male patients.^8^ To date, nearly 1000 cases have been well documented in the English literature. In the largest review reported to date, the age of patients ranged widely from 2 to 85 years with a mean age of 22 years, with a female to male ratio of about 10:1.^2^

Patients with SPT of the pancreas are often clinically asymptomatic. Some of them may present with a gradually enlarging abdominal mass or complain of vague abdominal pain or discomfort. Obstructive symptoms may occur earlier if the neoplasm is located in the pancreatic head. When a palpable mass is present, the average size of the tumor becomes quite remarkable (8–10 cm).^6^ SPTs have not been associated with specific clinical laboratory test findings or elevated serum pancreatic cancer markers. On gross examination, the mass is of variable size and well encapsulated and contains varying amounts of necrosis, hemorrhage, and cystic change. On light microscopic analysis, there are two distinct types of cellular arrangements: solid and papillary. The solid portions of the tumor are composed of uniform and polygonal epithelioid cells with well-vascularized stroma.^7^ Solid areas containing necrosis can be seen in most cases. The hallmark histological pattern occurs when the tumor cells form papillary configurations composed of a fibrovascular stalk surrounded by several layers of epithelial cells. Specific findings for SPT cells remain unclear and immunohistochemical staining is required for accurate diagnosis. Despite the fact that the phenotype of SPTs does not resemble that of any of the normal epithelial cells of pancreas, its immunoprofile is very distinctive. SPT cells are usually negative for endocrine markers, whereas they are characteristically positive for vimentin, CD10, AACT, AAT, β-catenin, NSE, Syn, and PRs (Fig. 4), all of which are very useful in differentiating them from endocrine pancreatic tumor cells.^9^

The exact cause of SPTs of the pancreas is unknown. Different hypotheses of histogenesis of SPTs have been postulated. Many investigators support the theory that SPTs originate from multipotent primordial cells, whereas others suggest that SPTs have an extra-pancreatic origin from genital ridge angle-related cells that were incorporated into the pancreas during organogenesis.^10^ Some support exists for both hypotheses. Many researchers have demonstrated that SPTs have a complex immunophenotype that is inconsistent with that of any of the pancreatic cell types and that a pancreatic origin is unlikely. On the basis of some similarities between SPTs and ovarian surface cells and the proximity between genital ridges and the pancreas anlage during early embryogenesis, SPTs might originate from the genital ridge-related cells that were incorporated into the pancreas during organogenesis.^10^ Although nearly all studies show no evidence of estrogen receptors, progesterone receptors are present in most cases.^8^ Furthermore, Morales et al.^11^ described the temporal relationship between a fast-growing SPT and pregnancy in a young woman. Hence, sex hormones may play a pivotal role in the pathogenesis or growth of SPTs. In addition, the low Ki67 expression in SPTs may be associated with a good clinical outcome of these tumors.

A variety of imaging techniques define the imaging features of SPTs as hypervascular, well-encapsulated round mass with combined cystic and solid components. USG findings of SPTs have been described as well-encapsulated cystic and solid masses. CT scan usually shows a well-encapsulated lesion with varying solid and cystic components owing to hemorrhagic degeneration.^12^ After intravenous contrast material administration, enhancing solid areas are typically noted peripherally, whereas cystic spaces are usually more centrally located.^13^ MR imaging typically shows a well-defined lesion with heterogeneous signal intensity on T1- and T2-weighted images, which reflects the complex nature of the mass. Areas of high signal intensity on T1-weighted images and low or inhomogeneous signal intensity on T2-weighted images can help identify blood products and may also help differentiate SPTs from other pancreatic tumors. Although recent advances of these imaging techniques have significantly improved pancreatic imaging, preoperative diagnosis of SPTs is still difficult. Accurate diagnosis of the special type of pancreatic tumor is obviously important. Fine needle aspiration or biopsy and histological analysis are needed for definitive diagnosis in certain cases.

## Conclusion

In conclusion, SPTs of the pancreas are a rare neoplasm comprising a wide range of differing underlying pathologies from completely benign through premalignant to frankly malignant. Surgical resection is the treatment of choice even in the case of distant hepatic metastasis, and complete resection is usually curative. The efficacy of chemotherapy or radiation therapy for SPTs has yet to be shown, although some cases were reported to be resectable after preoperative chemotherapy.^14,15^ The accumulation recognition and diagnosis of SPTs in males remains challenging.

## Abbreviations Used

AACT: α-1-antichymotrypsin
AAT: α-1-antitrypsin
CT: computed tomography
NSE: neuron-specific enolase
PR: progesterone receptor
SPT: solid pseudopapillary tumor
Syn: synaptophysin
USG: ultrasonography
